# Chlorophyll Fluorescence Imaging Uncovers Photosynthetic Fingerprint of Citrus Huanglongbing

**DOI:** 10.3389/fpls.2017.01509

**Published:** 2017-08-29

**Authors:** Haiyan Cen, Haiyong Weng, Jieni Yao, Mubin He, Jingwen Lv, Shijia Hua, Hongye Li, Yong He

**Affiliations:** ^1^College of Biosystems Engineering and Food Science, Zhejiang University Hangzhou, China; ^2^College of Agriculture and Biotechnology, Zhejiang University Hangzhou, China

**Keywords:** citrus, Huanglongbing, chlorophyll fluorescence imaging, photosynthesis, feature selection, classification

## Abstract

Huanglongbing (HLB) is one of the most destructive diseases of citrus, which has posed a serious threat to the global citrus production. This research was aimed to explore the use of chlorophyll fluorescence imaging combined with feature selection to characterize and detect the HLB disease. Chlorophyll fluorescence images of citrus leaf samples were measured by an in-house chlorophyll fluorescence imaging system. The commonly used chlorophyll fluorescence parameters provided the first screening of HLB disease. To further explore the photosynthetic fingerprint of HLB infected leaves, three feature selection methods combined with the supervised classifiers were employed to identify the unique fluorescence signature of HLB and perform the three-class classification (i.e., healthy, HLB infected, and nutrient deficient leaves). Unlike the commonly used fluorescence parameters, this novel data-driven approach by using the combination of the mean fluorescence parameters and image features gave the best classification performance with the accuracy of 97%, and presented a better interpretation for the spatial heterogeneity of photochemical and non-photochemical components in HLB infected citrus leaves. These results imply the potential of the proposed approach for the citrus HLB disease diagnosis, and also provide a valuable insight for the photosynthetic response to the HLB disease.

## Introduction

Huanglongbing (HLB), also known as greening disease, is one of the most destructive diseases to citrus industry. It is caused by a non-cultured phloem-restricted bacterium *Candidatus Liberibacter* with three different species, *Liberibacter africanus (Laf), Liberibacter asiaticus (Las)*, and *Liberibacter americanus (Lam)*, and is transmitted by the Asian citrus psyllid *Diaphorina citri* (Bové, 2006). HLB has threatened citrus production worldwide, including Asia, Africa, the United States, and Brazil. The infected trees are asymptomatic at early stages and become symptomatic anywhere from months to years varied with the cultivars. It could serve as a source of inoculums for pysllids with 60 days from the initial infection until it is infective (Lee et al., 2015), resulting in a rapid spread of infection in a glove of citrus trees. The leaf yellowing with blotchy mottle is considered as the most typical diagnostic symptom of HLB disease, especially on sweet oranges. The severely infected trees have smaller leaves with mottled patterns that are often similar to some nutrient deficient symptoms. In addition, the production of the HLB infected trees would dramatically decline with small and lopsided fruits, leading to the enormous economic loss.

So far, no effective control and cure measures are available for the HLB disease. There is an urgent need to develop an efficient method for the rapid and early detection of HLB disease in both field and laboratory conditions, and thereby in need of removal of the infected trees to prevent the further spread. The current commonly used HLB diagnosis involves experience-based inspection in the orchard and laboratory diagnostic tests. The former is usually performed by professional experts to recognize the typical symptoms of the disease in the field survey. Reported studies have shown that the accuracy of identifying an HLB infected tree by visual inspection is lower than 59% (Pourreza et al., 2015). Polymerase chain reaction (PCR) method that amplifies a DNA target sequence of the pathogen is usually employed as a standard laboratory-based diagnostic method. Although PCR can achieve a relative high accuracy for the HLB detection, it requires a time-consuming and labor-intensive sample preparation, and is not suitable for the continuous onsite detection and early disease warning. Therefore, a fast and non-destructive method is needed for growers to monitor their groves and control the spread of the disease.

Over the years, reflectance spectroscopy and imaging methods based on the selected or broad visible and near-infrared (VIS-NIR) electromagnetic wavelengths were investigated to detect plant diseases on the leaf and/or canopy levels (Inoue et al., 2012; Gnyp et al., 2014; Kusnierek and Korsaeth, 2015), and several studies have been reported on detection of citrus diseases. Qin et al. (2011) used two-band ratio images selected from the hyperspectral images to detect the canker disease of citrus fruit with the overall classification accuracy of 95.7%. Mishra et al. (2012) employed a VIS-NIR spectroscopy technique combined with three different classifiers for identifying HLB infected citrus trees. Although a reasonable classification accuracy was achieved by multiple spectral measurements of the tree canopy, the variation of sunlight and other environmental factors under real field conditions can add additional noises and reduce the detection accuracy. Later, they developed a vision sensor based on the polarization planar rotation of light by the starch at 591 nm, which improved the HLB detection accuracy in both zinc-deficient and non-zinc-deficient classes (Pourreza et al., 2014). However, the applicability of this method largely depends on the starch accumulation that varies in citrus cultivars and growing conditions (i.e., different orchards and seasonal factors). Sankaran et al. (2013) demonstrated the applicability of VIS-NIR and thermal imaging for detection of HLB disease in citrus trees, and they obtained an average overall accuracy of 87% for trees with symptomatic leaves. Li X. et al. (2012) applied multispectral and hyperspectral airborne imaging to detect HLB infected trees in citrus groves. However, the classification accuracies are relative low ranging from 28.7 to 90.2% by using different image classification methods due to the large positioning error of the ground truth. Garcia-Ruiz et al. (2013) compared two aerial imaging platforms for identifying HLB infected citrus trees with the classification accuracies in the range of 67–85% from unmanned aerial vehicle-based data, and 61–74% with aircraft-based data. In general, these results were not better than those from the ground-based remote sensing methods.

Fluorescence spectroscopy is also considered as a promising method for the rapid and early detection of biotic and abiotic stress response in plants. Fluorescence emissions from molecules of certain compounds such as plant pigments in leaves can be captured after the natural and artificial ultraviolet (UV) light excitation on a small sampling point (Pereira et al., 2011; Harbinson, 2013). Sankaran and Ehsani (2013) used a commercial handheld fluorescence sensor to collect fluorescence signal from healthy and HLB infected leaves of different citrus cultivars, and 97% classification accuracy in the discrimination of healthy and symptomatic HLB infected samples was achieved based on the bagged decision tree (BDT) classifier. However, the classification accuracy was reduced to about 81% when using the validation dataset from Hamlin and Valencia samples, and the overall accuracy of differentiating healthy from asymptomatic HLB infected samples was also very low with the best result of 48.2% when using the support vector machine. Due to the limitation of the sampling area, the spatial heterogeneity in a leaf caused by plant diseases or other physiological disorders may not be accurately described. Researchers proposed that the fluorescence imaging technique could be more useful in detecting the plant physiological response since it can obtain the fluorescence signal as well as the within-plant variation (Pinto et al., 2016; Song et al., 2016). Different fluorescence imaging techniques with different excitation modes have been developed. Steady-state UV light-induced fluorescence imaging has been used to estimate anthocyanin in strawberries and visualize systemic viral infections in *Nicotiana benthamiana* plants by monitoring the signals of the chlorophyll fluorescence and blue-green fluorescence, respectively (Pineda et al., 2008a; Yoshioka et al., 2013). Fluorescence imaging spectroscopy with 530 nm excitation that can obtain a fluorescence image at 690 nm, was also employed to discriminate symptomatic HLB infected leaves from healthy ones sampling from two different orchards (Wetterich et al., 2013). Results showed a good accuracy (90%) for Brazil samples but low (61%) for United States samples. Later, they applied another two excitation sources (405 and 470 nm) to detect citrus HLB disease from nutrient deficient leaves, as well as healthy ones (Wetterich et al., 2016, 2017). The combination of fluorescence bands from two excitations improved the classification accuracy in the range of 92–95% when discriminating HLB from zinc-deficiency by using different machine learning methods. However, more detailed knowledge about energy partition in photosystem II (PSII) should be understood in HLB infected leaves. Kinetic chlorophyll fluorescence imaging provides an efficient way to trace energy partitioning in the photosystem II (PSII) and monitor the electron transport pattern in photosynthesis. It plays an important role in understanding the fundamental mechanism of photosynthesis, pathology, and phenotypic plasticity to genetic variations and plant environmental changes (Murchie and Lawson, 2013; Choi et al., 2016; Sui et al., 2017). Kinetic chlorophyll fluorescence imaging has been widely used to evaluate freeze-thaw and drought tolerance in *Arabidopsis* (Ehlert and Hincha, 2008; Bresson et al., 2015), salt stress response in wheat (Mehta et al., 2010), chronic ozone damage to soybean leaves (Chen et al., 2009), and plant virus infection (Pineda et al., 2011; Lei et al., 2017). However, few studies have used kinetic chlorophyll fluorescence imaging to study the HLB disease effects on plant photosynthetic functions and complex pathogenesis. Furthermore, the spatial heterogeneity during the disease development implies additional challenges for the rapid diagnosis of HLB disease.

Therefore, this research was aimed to characterize the photosynthetic function of the HLB infected leaves by measuring the fluorescence signals using kinetic chlorophyll fluorescence imaging, and extract the photosynthetic fingerprints that can uniquely identify the HLB disease by performing the advanced machine learning and statistical analysis. The findings in this research provide an important insight to understand the citrus HLB disease infection related to changes in plant photosynthetic activities, and demonstrate that kinetic chlorophyll fluorescence imaging could offer a rapid and non-invasive means for detecting HLB in citrus trees.

## Materials and Methods

### Leaf Sample Collection

Citrus leaf sampling was carried out at a commercial orchard in Nanping, Fujian Province, China in March 2016. The branches were detached from the citrus trees from three categories including healthy, HLB infected (symptomatic and asymptomatic), and nutrient deficient (zinc and magnesium deficient) samples marked by the HLB experts, and immediately wrapped with wet cottons and placed inside a cooler to avoid desiccation. Leaves were then detached from the branches in the laboratory (Supplementary Figure S1), and chlorophyll fluorescence image acquisition was performed immediately after detachment.

### Kinetic Chlorophyll Fluorescence Imaging

Chlorophyll fluorescence images of leaf samples were measured by an in-house chlorophyll fluorescence imaging system (FluorCam FC800, Photon Systems Instruments, Brno, Czechia) (Supplementary Figure S2) after dark adaptation. A CCD camera with a prime lens (SV-H1.4/6, VS Technology, Tokyo, Japan) was used to capture chlorophyll fluorescence transients in a batch of images with the spatial resolution of 696 × 520. Four light-emitting diodes (LEDs) panels with the incident angle of 45° were installed as the light source, which include two red-orange LEDs (620 nm) panels (<0.1 μmol⋅m^-2^⋅s^-1^) for flashes and actinic light 1 (0–250 μmol⋅m^-2^⋅s^-1^), and two cool white LEDs (>8000 K) panels (0–1600 μmol⋅m^-2^⋅s^-1^) for actinic light 2 and saturating flashes (0–3000 μmol⋅m^-2^⋅s^-1^). A leaf sample holder with a manually adjustable vertical stage was used for positioning samples to an imaging distance of 20 cm from the lens.

**FIGURE 1:**
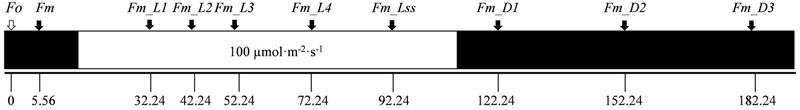
Schematic description of the chlorophyll fluorescence quenching protocol in this study. Dark boxes indicate the dark-adapted periods during the measurement. The white box is related to the irradiance of actinic light (100 μmol⋅m^-2^⋅s^-1^). Solid black arrows represent the moments of irradiance of saturating flashes (800 μmol⋅m^-2^⋅s^-1^), and the hollow arrow indicates the state of *Fo* measurement.

Before the experiment, a preliminary study was performed to determine the optimal protocol for the kinetic chlorophyll fluorescence image acquisition of citrus leaves. The dark-adapted time was determined by checking the value of the maximum PSII quantum yield (*Fv/Fm*) of citrus leaves at different dark-adapted time as shown in Supplementary Figure S3. It was observed that *Fv/Fm* was stable with the value of 0.80 after 20 min dark adaptation, which was considered as the optimal time. Although this 20 min period may be a problem for practical applications, it is appropriate for achieving this research goal. In addition, the intensity of the saturating pulse was described in percentage in image acquisition software. 50% was considered as the optimal intensity of the saturating pulse when the value of *Fv/Fm* reached 0.81 (Supplementary Figure S4), which corresponded with the absolute intensity value of 800 μmol⋅m^-2^⋅s^-1^ when measured at the position of 20 cm distance from the lens using a quantum meter (Model MQ-200, Apogee Instruments, Inc. United States). The detailed chlorophyll fluorescence quenching protocol is described in **Figure 1**. The minimum fluorescence in dark-adapted state (*Fo*) was measured after 20 min dark adaptation, and followed by a strong flash of light of 800 μmol⋅m^-2^⋅s^-1^ at 5.56 s that transiently reduces the plastoquinone pool and the primary quinone acceptor *Q*_A_ so that the maximum fluorescence in dark-adapted state (*Fm*) was recorded. The leaf sample was then exposed to an actinic light with the intensity of 100 μmol⋅m^-2^⋅s^-1^ for 70 s. Saturating flashes (800 μmol⋅m^-2^⋅s^-1^) were applied to measure the maximum fluorescence during light adaptation (*Fm_Ln*) at 32.24, 42.24, 52.24, and 72.24 s, respectively, and the maximum fluorescence at the light-adapted steady state (*Fm_Lss*) was obtained during the saturating flash (*t* = 92.24 s) at the end of the actinic light period. The instantaneous maximum fluorescence signals during dark relaxation (*Fm_Dn*) with the saturating flashes at 122.24, 152.24, and 182.24 s were also measured. Based on the measured fluorescence signals, the variable fluorescence in dark-adapted state (*Fv* = *Fm - Fo*), instantaneous non-photochemical quenching during light adaptation (*NPQ_Ln* = (*Fm - Fm_Ln)/Fm_Ln*), steady-state non-photochemical quenching (*NPQ_Lss* = (*Fm - Fm_Lss)/Fm_Lss*), and instantaneous non-photochemical quenching during dark relaxation (*NPQ_Dn* = (*Fm - Fm_Dn)/Fm_Dn*) can be also obtained.

### PCR Analysis

Polymerase chain reaction analysis was conducted to confirm the HLB status of leave samples at Plant Pathology Laboratory of Zhejiang University. Two sets of primers designed from conserved regions of 16S ribosomal DNA were synthesized for HLB detection at TSINGKE Biological Technology, China. DNA was extracted from the healthy, nutrient deficient, and HLB infected leaf samples using CTAB (Cetyltrimethylammonium Bromide) methods with the following protocol, respectively: 200 mg midrib tissue of each leaf was transferred to a 2 mL centrifuge tube and ground to the fine powder in liquid nitrogen using an automatic grinding miller (Tissuelyser-64, Shanghai Jingxin Industrial Development Co., Ltd.). 800 μL CTAB was then added into the tube, and heated at 65°C in water bath for 30 min with intermittent agitation. 800 μL mixture (phenol: chloroform: isopropanol = 25: 24: 1) was added and the tube was centrifuged at 12,000 rmp for 10 min at room temperature. 500 μl supernatant was then transferred into a new 2 mL tube, and 1000 μL 95% alcohol and 150 μL NaAC were added. The homogenate was placed on the ice for 12 min and then centrifuged at 12,000 rmp for 2 min. DNA was re-precipitated with 400 μL of 75% alcohol at 12,000 rmp for 2 min. 100 μL ddH_2_O was added to the precipitation and DNA was stored at -20°C at last. PCR amplification was conducted in a 20 μL mixtures reaction using 7.5 μL ddH_2_O, 1 μL Primer-F, 1 μL Primer-R, 10 μL green taq mix (Vazyme) and 0.5 μL DNA template. The detailed PCR amplification protocol can be found in the literature (Gouda et al., 2006).

### Data Analysis

A total of 26 images for each leaf sample related to the fluorescence quenching process were obtained from kinetic chlorophyll fluorescence imaging, which provide detailed information of dynamics of PSII activities about plant status. Commonly used fluorescence parameters such as *Fv/Fo, Fv/Fm*, and *NPQ_Lss* can be easily calculated by averaging the intensity of the region of interest (ROI) for the corresponding fluorescence image. They are considered as the important parameters in the analysis of photosynthesis associated with plant physiological changes, and their capabilities of identifying the HLB infected leaves from the healthy or nutrient deficient leaves were investigated by advanced machine learning and statistical analysis. Feature selection methods were performed on all the fluorescence parameters to extract the unique fluorescence features that could develop a photosynthetic fingerprint of the HLB disease. Three feature selection methods including random frog (RF), sequential forward selection (SFS), and Monte Carlo uninformative variable elimination (MC-UVE) were used. RF algorithm is based on the reversible jump Markov Chain, and its output provides the selection probability of each feature that can be used as a measure of feature importance. The detailed information of RF can be found in the literature (Li H. et al., 2012). The SFS algorithm is a bottom-up process that starts with an empty feature subset and repeatedly adds the most significant feature selected by an objective function. The feature cannot be discarded at a later stage once it is retained. The fisher criterion is usually used as an objective function (Geng, 2014). MC-UVE develops multiple models with randomly selected calibration sample set produced by the MC method, and the variable is then evaluated with the stability of the corresponding coefficient of the model (Han et al., 2008).

The development of the HLB disease and the spread of the symptoms vary spatially in the leaf, which result in a large variation of fluorescence intensities in the image. Therefore, image features were also investigated for the further classification. Principal component analysis (PCA) was applied to reduce the dimension of the chlorophyll fluorescence image cubes and obtain an uncorrelated orthogonal basis set from the original image set. Scale-invariant feature transform (SIFT) was then used to detect the local features in principal component images. The feature descriptors were first computed as orientation histograms and then transformed to a 128 dimensional SIFT feature vector, which is invariant to the image scale and rotation and robust to local geometric distortions.

Both the mean fluorescence parameter-based and image-based classification models were developed for detecting the HLB disease, and the inputs of the models were the features extracted from the mean fluorescence parameters and the principal component images, respectively. Two classifiers including partial least squares discriminant analysis (PLS-DA) and support vector machine (SVM) were employed to discriminate the HLB infected leaves from the healthy and the nutrient deficient ones. The PLS-DA algorithm is an extension of the PLS model, where the dependent variable is a vector that represents the class label values for each class (Aliakbarzadeh et al., 2016). The SVM classifier is developed based on the statistical learning theory to find a hyperplane that gives the largest distance between the margins of the training data set, and it can be achieved by solving a convex quadratic programming problem using a kernel function (Chapelle et al., 2002; Cen et al., 2016). The three-class classification was performed by labeling “1” for healthy samples, “2” for HLB infected samples and “3” for nutrient deficient samples. All the samples were divided into two groups with 60 for the training set and 30 for the validation set by using Kennard-Stone algorithm (Macho et al., 2001). The machine learning and the statistical analysis were performed using Matlab 2011a (The Mathworks, Inc., Natick, MA, United States) and IBM SPSS Statistics (version 20.0, IBM Corporation, Armonk, NY, United States).

## Results

### PCR Result

**FIGURE 2:**
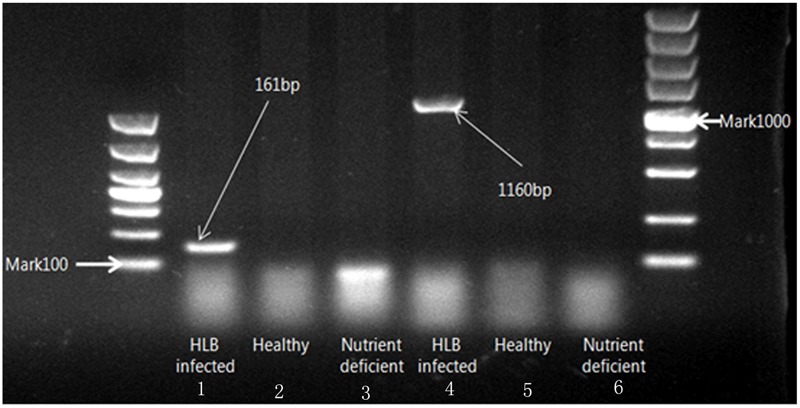
Electrophoresis on 1% agarose gel of DNA amplified with two specific primers (OI1/OI2c and rplLAS-F/rplLAS-R).

The status of leaves was verified though amplifying target DNA template obtained from leaf midrib tissue. The sensitivity of PCR analysis with different primer sets is shown in **Figure 2**. Two different sizes of amplicons (1160 and 161 bp) from the 16S ribosomal DNA represented the HLB bacterium DNA template amplified with two specific primers (OI1/OI2c and rplLAS-F/rplLAS-R), respectively. It is indicated that lanes 1 and 4 were PCR positive, and lanes 2, 3, 5, and 6 were PCR negative. Finally, citrus samples were divided into three classes with 30 healthy, 30 HLB infected, and 30 nutrient deficient samples.

### Effect of HLB Infection on Commonly Used Chlorophyll Fluorescence Parameters

**FIGURE 3:**
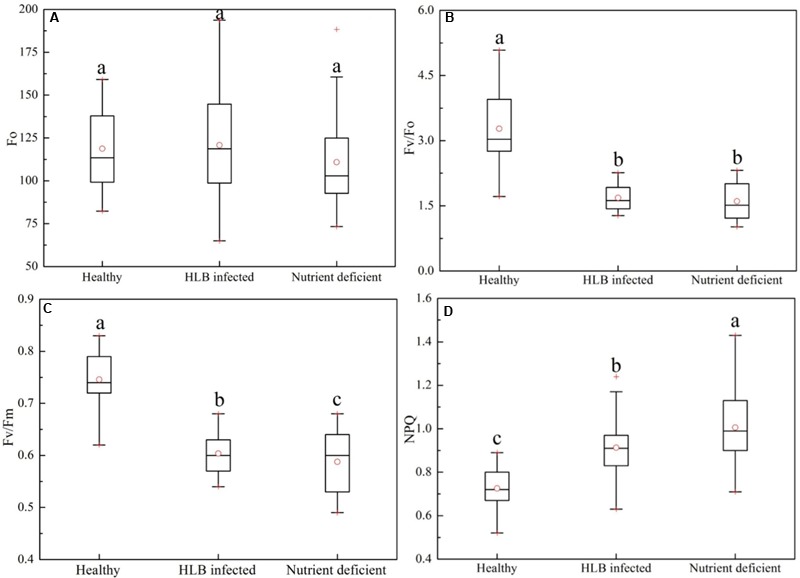
Box-and-whisker plots of commonly used chlorophyll fluorescence parameters including **(A)** *Fo*, **(B)** *Fv/Fo*, **(C)** *Fv/Fm*, and **(D)** *NPQ* of healthy, HLB infected and nutrient deficient leaves. Different letters indicated significant differences (p < 0.05) by using Duncan test. Values in the figure were mean ± standard deviation (SD).

**Figure 3** shows a statistical summary (i.e., mean, range, median, outliers, and quartiles) of four commonly used chlorophyll fluorescence parameters, *Fo, Fv/Fo, Fv/Fm*, and *NPQ_Lss*, in healthy, HLB infected, and nutrient deficient leaves. There was no statistically significant difference in *Fo* among the three classes (**Figure 3A**); while the variation of *Fo* in the HLB infected leaves was larger than those in healthy and nutrient deficient samples. Compared with healthy leaves, *Fv/Fo* decreased by 48.9 and 51.1% in HLB infected and nutrient deficient leaves, respectively, indicating the decreased photosynthetic rate in unhealthy leaves. However, no difference in *Fv/Fo* between HLB infected and nutrient deficient classes was observed (**Figure 3B**). The parameters *Fv/Fm* and *NPQ_Lss* showed significant differences among healthy, HLB infected and nutrient deficient leaves (**Figures 3C,D**). *Fv/Fm* of healthy samples was higher than those of other two classes, and *Fv/Fm* of HLB infected samples decreased less than those in nutrient deficient ones (**Figure 3C**). The values of *NPQ_Lss* in HLB infected and nutrient deficient leaves increased compared with those in healthy leaves. This result provided the first screening of HLB disease using the commonly used chlorophyll fluorescence parameters.

**FIGURE 4:**
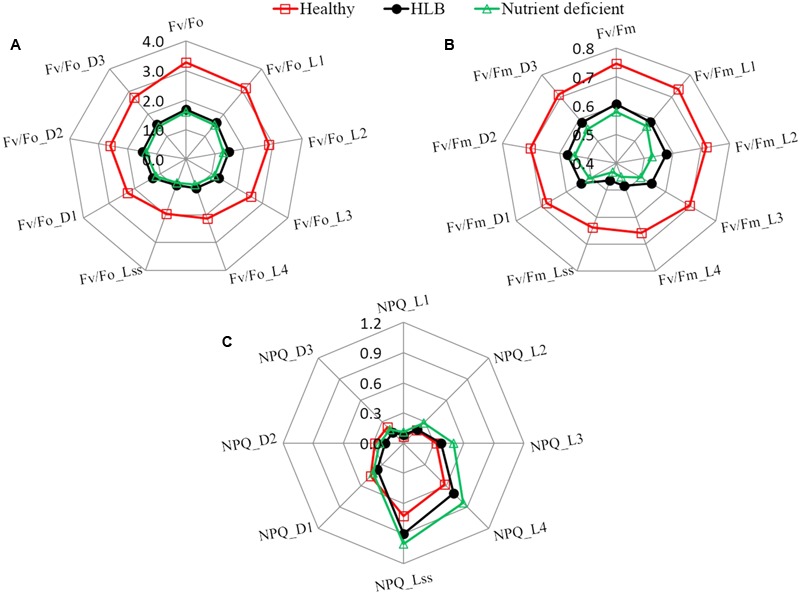
Spider plots of selected fluorescence parameters including **(A)** *Fv/Fo*, **(B)** *Fv/Fm*, and **(C)** *NPQ* at different states of healthy, HLB infected and nutrient deficient citrus leaves measured during fluorescence quenching process.

Furthermore, a set of chlorophyll fluorescence quenching parameters (*Fv/Fo_Ln, Fv/Fm_Ln, NPQ_Ln, Fv/Fo_Dn, Fv/Fm_Dn*, and *NPQ_Dn*) related to the stress-induced changes in the photosynthetic process were analyzed using the spider plots as shown in **Figure 4**. It includes the parameters measured at the dark-adapted state, light adaptation, and dark relaxation. The distance from the center of the spider plot indicates the relative change of the fluorescence parameter with different leaf conditions. Generally, fluorescence quenching parameters of HLB infected and nutrient deficient leaves exhibited a strong contrast compared with those of healthy samples, which is consistent with the reported studies (Pineda et al., 2008b; Spoustová et al., 2013; Mishra et al., 2016). The instantaneous variable-to-initial fluorescence and the maximal PSII quantum yield during light adaptation (*Fv/Fo_Ln* and *Fv/Fm_Ln*) and dark relaxation (*Fv/Fo_Dn* and *Fv/Fm_Dn*) dramatically decreased in HLB infected and nutrient deficient leaves. The difference in instantaneous maximal PSII quantum yield at multiple phases between the HLB infected and nutrient deficient samples was also observed. The value of instantaneous non-photochemical quenching in HLB infected and nutrient deficient leaves during light adaptation increased significantly, particularly, a large difference between the HLB infected and the nutrient deficient leaves was observed during the third (*NPQ_L3*) and fourth (*NPQ_L4*) of the saturating flashes and the light-adapted steady-state (*NPQ_Lss*). Generally, the results shown in **Figures 3, 4** reveal that commonly used fluorescence parameters have the potential for identifying the unhealthy leaves from the healthy ones, but it might be a challenge to differentiate the HLB infected leaves from the nutrient deficient ones by only using these parameters.

### HLB Detection Based on Optimal Features Extracted from Mean Fluorescence Parameters

**FIGURE 5:**
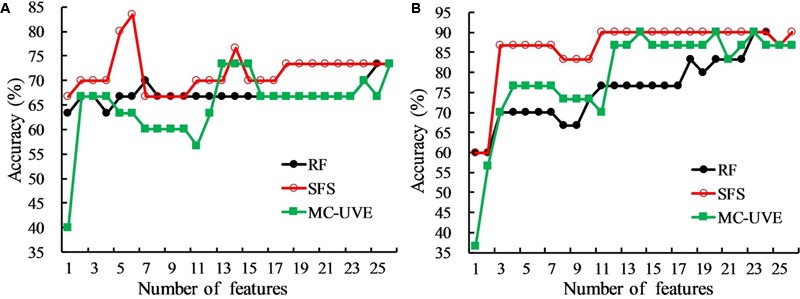
Comparison of classification accuracies of random frog (RF), sequential forward selection (SFS), and Monte Carlo of uninformative variables elimination method (MC-UVE) using **(A)** partial least squares discriminant analysis (PLS-DA) and **(B)** support vector machine (SVM) classifiers for the classification of healthy, HLB infected, and nutrient deficient leaves.

Commonly used chlorophyll fluorescence parameters have demonstrated that both HLB infection and nutrient deficiency could cause the change of fluorescence emission as presented in **Figures 3, 4**. However, they may not be the best parameters that would provide an accurate detection of HLB disease. Feature selection methods were applied here to extract the optimal features from all the mean fluorescence parameters that could characterize HLB infected leaves, and their performances are shown in **Figure 5**. It was observed that the classification accuracies varied greatly with the number of selected features, and exhibited the increasing tendency with the increased feature number. In general, the SFS selected features clearly exceeded those obtained from RF and MC-UVE methods when using both SVM and PLS-DA classifiers for three-class classification; while the performance of the RF and MC-UVE was more influenced by the classifier. As shown in **Figure 5B**, SFS-SVM achieved the best classification result of 90% with the feature subset size of 11, denoting that these 11 features might involve the most important fluorescence signatures related to the photosynthesis process affected by the HLB disease and nutrient deficiency, resulting in a better classification among healthy, HLB infected, and nutrient deficient leaves. It was also noted that the classification performance with selected features by SFS was better than that with the full dataset.

The best classification results using three feature selection methods with two classifiers based on mean fluorescence parameters are presented in **Table 1**. The SVM classifier significantly outperformed PLS-DA with the classification accuracy of 90% when using the features from all three feature selection methods. Among the combinations of the two classifiers and three feature selection methods, SFS with each of the two classifiers tended to produce the smallest feature subsets of 6 for PLS-DA and 11 for SVM with the high classification accuracies. The result also showed that the non-photochemical quenching related parameters measured during light adaptation were all selected by three methods, which was consistent with the previous analysis in **Figure 4C**.

**Table 1:** Classification results based on chlorophyll fluorescence parameter analysis with the optimal features.

Feature	First three	Classifier	Feature	Healthy (%)	HLB infected (%)	Nutrient	Overall
selection	selected features		number			deficient (%)	accuracy (%)
RF	*Fv/Fm_D3, NPQ_L3, Fv/Fo_D2*	PLS-DA	7	80	80	50	70
		SVM	23	80	90	100	90
SFS	*Fv/Fm_Lss, NPQ_Lss, NPQ_L2*	PLS-DA	6	90	80	80	83
		SVM	11	80	90	100	90
MC-UVE	*NPQ_L3, Fv/Fm_D3, Fv/Fo_L4*	PLS-DA	13	90	80	50	73
		SVM	23	80	90	100	90

*RF, random frog; SFS, sequential forward selection; MC-UVE, Monte Carlo of uninformative variables elimination method; PLS-DA, partial least squares discriminant analysis; SVM, support vector machine.*

### Chlorophyll Fluorescence Image-Based Classification

**FIGURE 6:**
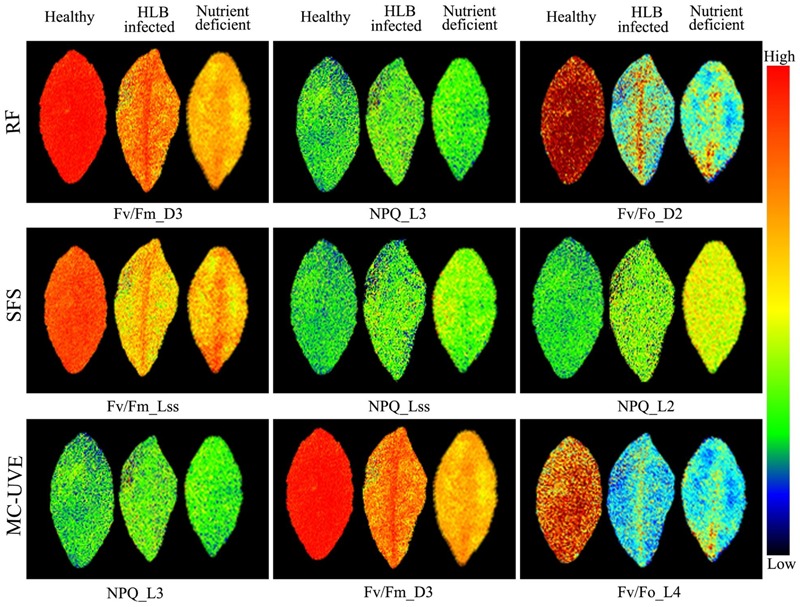
Images of representative samples of first three features selected by RF, SFS, and Monte Carlo of uninformative variables elimination (MC-UVE).

Chlorophyll fluorescence images related to the first three optimal parameters selected by RF, SFS, and MC-UVE methods were shown in **Figure 6**. The variations among healthy, HLB infected and nutrient deficient leaves were clearly observed. Some parameter images also showed the heterogeneity within the leaf due to the HLB infection or nutrient deficiency. This is agreement with the reported study on a localized decrease in photosynthesis during the disease infection or other physiological disorder processes (Kenny et al., 2011; Calatayud et al., 2013).

The spatial heterogeneity of the HLB symptoms caused by the HLB bacterium presents more challenges for the disease identification. Hence, chlorophyll fluorescence image-based analysis was also performed to improve the HLB detection accuracy. The first six principal component (PC) images representing over 95% variables were used for image feature extraction. Features extracted by SIFT from PC images were used as the SVM classifier input. **Table 2** summarized the three-class classification results of using SVM based on image features and image features combined with the mean fluorescence parameters, respectively. The overall accuracy based on image features was 77%, which was not as good as that using mean fluorescence parameters with the classification accuracy of 90%. However, the combination of the image features and the mean fluorescence parameters significantly improved the classification performance with the overall accuracy of 97%. This suggests that contrast information among these three classes was enhanced when using data fusion.

**Table 2:** Support vector machine (SVM) classification results based on the principal component (PC) image features and the combination of image features and mean fluorescence parameters.

Sample status	PC images			Combination of PC image features		
	features			and mean fluorescence parameters		
	Healthy	HLB infected	Nutrient deficient	Healthy	HLB infected	Nutrient deficient
Healthy	8 (80%)	2	0	10 (100%)	0	0
HLB infected	0	7 (70%)	3	0	9 (90%)	1
Nutrient deficient	0	2	8 (80%)	0	0	10 (100%)
Overall accuracy		77%			97%

## Discussion

Various studies have reported that chlorophyll fluorescence is a promising technique for non-invasive measurement of PSII activities, and could achieve a rapid screening of photosynthetic processes related to the plant status (Ehlert and Hincha, 2008; Rolfe and Scholes, 2010; Ivanov and Bernards, 2016). Commonly used chlorophyll fluorescence parameters, such as *Fo, Fv/Fo, Fv/Fm*, and *NPQ_Lss*, obtained by exposing the leaf to a combination of darkness, actinic lights and a series of saturating flashes as shown in **Figure 1**, are considered as the most useful parameters to interpret energy dissipation of chlorophyll in the thylakoid membrane. In our study, these four fluorescence parameters measured in healthy, HLB infected, and nutrient deficient leaves showed the potential of differentiating the unhealthy leaves from the healthy ones except *Fo* (**Figure 3**). Large variations in *Fo* might be one of the reasons that it is difficult to provide a straightforward explanation about the impacts of HLB infection and nutrient deficiency on the PSII reaction center and energy transfer. The parameter *Fv/Fo* is considered as an indicator of the number and the size of active photosynthetic reaction centers (Dan et al., 2000). The dramatic declines of *Fv/Fo* in HLB infected and nutrient deficient leaves indicate the change in the rate of electron transport from PSII to the primary electron acceptors with the reduction of the number and the size, which have also been reported in different plants exposed to the disease and the environmental stresses (Zhou et al., 2009; Martinazzo et al., 2012; Janka et al., 2013). The decrease of *Fv/Fm* in HLB infected and nutrient deficient leaves could be a result of the major damage to the photosynthetic apparatus in response to the HLB infection and nutrient deficiency. The value of *NPQ_Lss* in HLB infected and nutrient deficient leaves might be related to the different degrees of tissue damage, which can also be reflected from the severity of the HLB and nutrient deficient symptoms. Reported studies have revealed that the increase of the *NPQ_Lss* depends on the stimulated electron flow as a protection mechanism (Tatagiba et al., 2016). What’s more, it indicates the excess excitation energy dissipated as heat in order to reduce the photooxidative damage, which is also coupled to a gradual loss of chloroplastic pigments in response to the HLB infection and nutrient deficiency, eventually resulting in blotchy yellowing symptoms in leaves. Meanwhile, the significant differences observed in *Fv/Fm* and *NPQ_Lss* values between the HLB infected and nutrient deficient leaves demonstrated that it was possible to identify HLB infected leaves from the nutrient deficient ones, although the interpretation is not straightforward for a number of reasons.

Although previous research has reported a desired discrimination accuracy (92–95%) with adding fluorescence at 690 nm by using fluorescence imaging spectroscopy suggesting the significant contribution of emission from chlorophyll *a* molecule for HLB detection (Wetterich et al., 2016, 2017), more fundamental understanding about energy partition in PSII in the HLB infected leaves should be gained. The chlorophyll fluorescence quenching process represents the complex dynamics of plant photosynthetic reaction during a transition from a dark-adapted to a light-adapted state (Govindjee, 1995). Some parameters measured at different time-courses during quenching process could offer photosynthetic signatures related to the plant disease (Berger et al., 2007). Further analysis of chlorophyll fluorescence parameters obtained during fluorescence quenching process as shown in **Figure 4** provided additional feature information related to the HLB disease, especially the consistent decrease in instantaneous non-photochemical quenching at several states in response to the HLB infection indicated a direct effect of HLB disease on PSII due to the irreversible damage of the leaf tissue.

Advanced machine learning and statistical analysis of finding the photosynthetic fingerprints of the HLB disease detection was shown to be superior to the commonly used fluorescence parameters. Designing an effective criterion to select an optimal subset of features is a challenging problem for data classification. For the mean fluorescence parameters analysis, SFS-SVM reached a best overall accuracy of 90% suggesting that features selected by SFS can better describe the pattern of the fluorescence data than the other two methods. Compared with the commonly used fluorescence parameters, feature selection methods provided a new insight to achieve a better interpretation of the original data by removing the redundant information and enhancing the most useful information related to the HLB infected characteristics. Although image features could provide the spatial information about the photochemical and non-photochemical components, the performance of the three-class classification based on image features extracted from PC images combined with the SVM classifier, was not better than those from mean fluorescence parameters (**Tables 1, 2**). This suggests that nutrient deficiency in leaves would induce similar image texture to HLB infected ones as shown in **Figure 6**. This is probably a reason why local image features had a poorer classification performance. The best classification accuracy of 97% using the combination of image features and mean fluorescence parameters revealed its capability to discriminate among healthy, HLB infected, and nutrient deficient leaves. In general, the results obtained in this study was comparable to those obtained using other spectroscopy and imaging techniques (Garcia-Ruiz et al., 2013; Sankaran et al., 2013; Wetterich et al., 2016, 2017). However, one should notice the differences of experimental conditions and samples when comparing our classification accuracy with those from other techniques. It is true that the conventional method depending on the commonly used chlorophyll fluorescence parameters provided a better plant physiological interpretation, but it is challenging to perform a quantitative analysis of decision making for the HLB diagnosis in various conditions. Recent studies have approved that feature selection methods as well as data fusion have exploited a new way to interpret and extract the hidden information from the original data, such as image and spectral data (Li et al., 2015; Liu and Li, 2016; Fu et al., 2017). Better classification results by employing proper feature selection method and classifier (**Table 2**) demonstrated the capability of this data-driven strategy for the chlorophyll fluorescence image analysis.

In this study, we presented a comprehensive investigation on the photosynthetic characteristics of HLB infected citrus leaves and the possibility of the HLB disease detection using chlorophyll fluorescence imaging combined with feature selection. The combination of mean fluorescence parameters and images features significantly improved the classification performance with the accuracy of 97%, which indicated a better interpretation for the spatial heterogeneity of photochemical and non-photochemical components in HLB infected citrus leaves. The proposed approach cannot only achieve a better detection accuracy of the HLB disease, but also be developed as a new means for the plant photosynthetic analysis.

## Author Contributions

HC and HW designed the experiment. HW, JY, MH, JL, and SH performed the experiment. HC and HW contributed to the data analysis and wrote the manuscript. HL and YH provided suggestions on the experiment design and discussion sections.

## Conflict of Interest Statement

The authors declare that the research was conducted in the absence of any commercial or financial relationships that could be construed as a potential conflict of interest.
