# Supplementary material for: Chlorophyll Fluorescence Imaging Uncovers Photosynthetic Fingerprint of Citrus Huanglongbing
Source: Front Plant Sci. 2017 Aug 29;8:1509. doi: 10.3389/fpls.2017.01509 (PMC5581828; doi:10.3389/fpls.2017.01509)
Supplement: Supplementary file 1 [file Data_Sheet_1.DOCX]

Supplementary Material

**Chlorophyll fluorescence imaging uncovers photosynthetic fingerprint of citrus Huanglongbing**

Haiyan Cen^1^, Haiyong Weng^1^, Jieni Yao^1^, Mubin He^1^, Jingwen Lv^2^, Shijia Hua^1^, Hongye Li^2^, Yong He^1*^

*** Correspondence:**Yong He
[yhe@zju.edu.cn](mailto:yhe@zju.edu.cn)

# Supplementary figures

There were three categories of leaf samples in this investigation: healthy, HLB infected (symptomatic and asymptomatic), and nutrient deficient (zinc and magnesium deficient). The branches were cut from trees, and immediately wrapped with wet cottons and placed inside a cooler to avoid desiccation. Leaves were then detached from the branches in the laboratory, and chlorophyll fluorescence image acquisition was performed immediately after detachment.

**
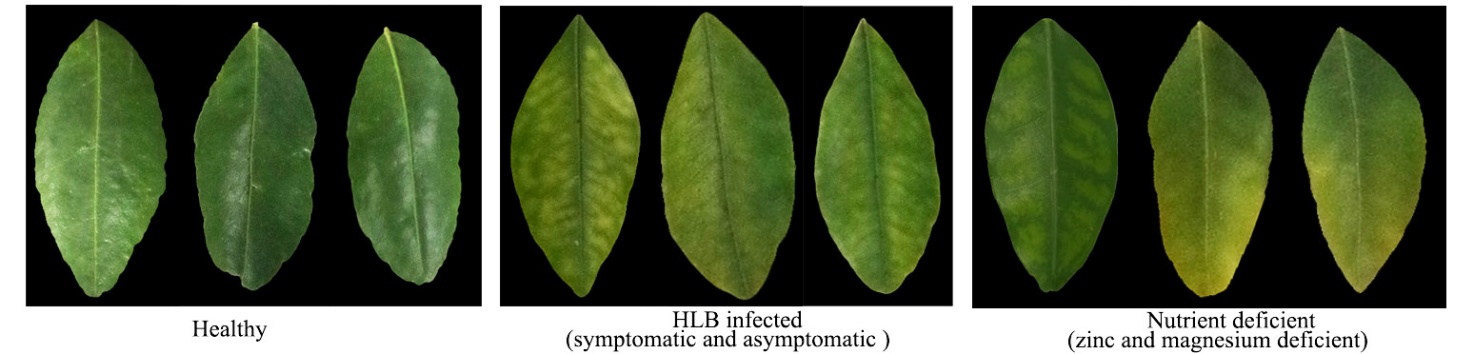
**

Figure S1. Representative RGB images of healthy, HLB infected (symptomatic and asymptomatic) and nutrient deficient (zinc and magnesium deficient) leaves.

A in-house chlorophyll fluorescence imaging system (FluorCam FC800, Photon Systems Instruments, Brno, Czech Republic) used in our research includes a CCD camera with a prime lens (SV-H1.4/6, VS Technology, Tokyo, Japan) to capture chlorophyll fluorescence transients in a batch of images with the spatial resolution of 696🞨520; Four light-emitting diodes (LEDs) panels with the incident angle of 45° were installed as the light source, which include two red-orange LEDs panels (<0.1 µmol·m^-2^·s^-1^) for flashes and actinic light 1 (0-250 µmol·m^-2^·s^-1^), and two cool white LEDs panels (0-1600 µmol·m^-2^·s^-1^) for actinic light 2 and saturating flashes (0-3000 µmol·m^-2^·s^-1^); A leaf sample holder with a manually adjustable vertical stage was used for positioning samples to a suitable position.

**
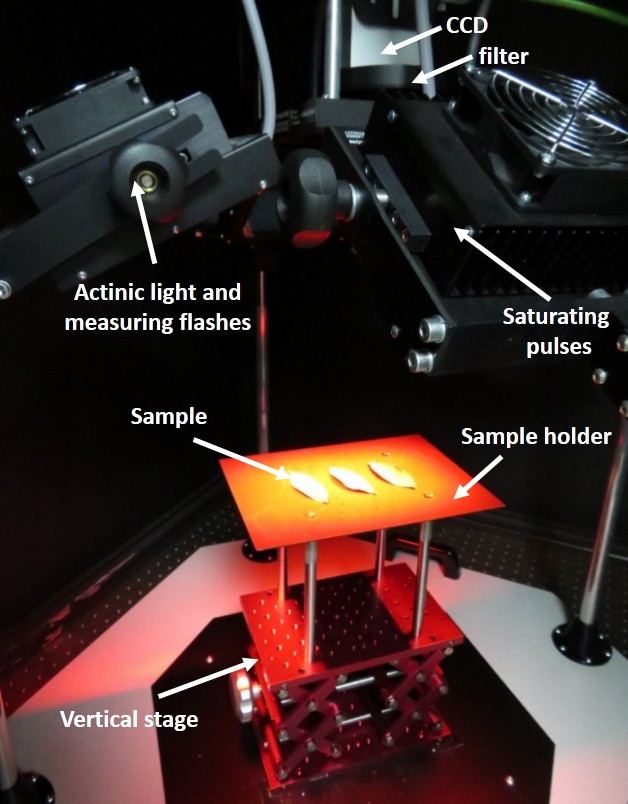
**

Figure S2. Setup of the kinetic chlorophyll fluorescence imaging system.

Before the experiment, we performed a preliminary study to determine the optimal protocol (i.e., dark-adapted time, dark relaxation time, light adaptation time, saturating flashing time, intensity of saturating flashes, incident angle of the light source) for the kinetic chlorophyll fluorescence image acquisition of citrus leaves. The dark-adapted time was determined by checking the value of the maximum PSII quantum yield (*Fv/Fm*) of citrus leaves at different dark-adapted time as shown in Figure S3. It was observed that (*Fv/Fm*) was stable with the value of 0.80 after 20 min dark-adapted, which was suitable for our study.

**
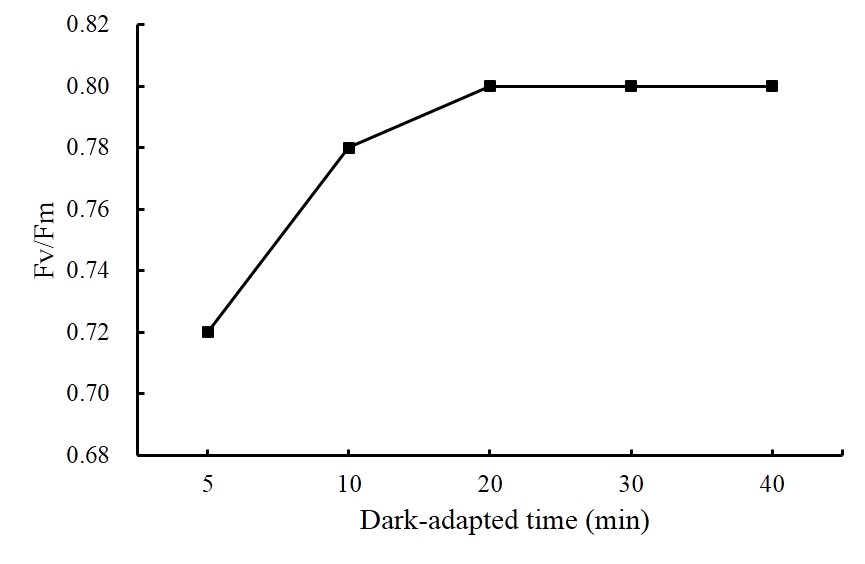
**

Figure S3. Effect of dark-adapted time on the maximum PSII quantum yield (*Fv/Fm*) of a healthy citrus leaf.

In this study, the intensity of the saturating pulse was described in percentage in the software provided by PSI manufacture. 50% was considered as the optimal intensity of the saturating pulse when the value of Fv/Fm reached 0.81, which corresponded with the absolute intensity value of 800 µmol·m^-2^·s^-1^ when measured at the position of 20 cm distance from the lens using a quantum meter (Model MQ-200, Apogee Instruments, Inc. USA). Therefore, the absolute intensity mentioned here really depends on the position where to measure. We also measured the intensity close to the light source, it reached 3000 µmol·m^-2^·s^-1^.

**
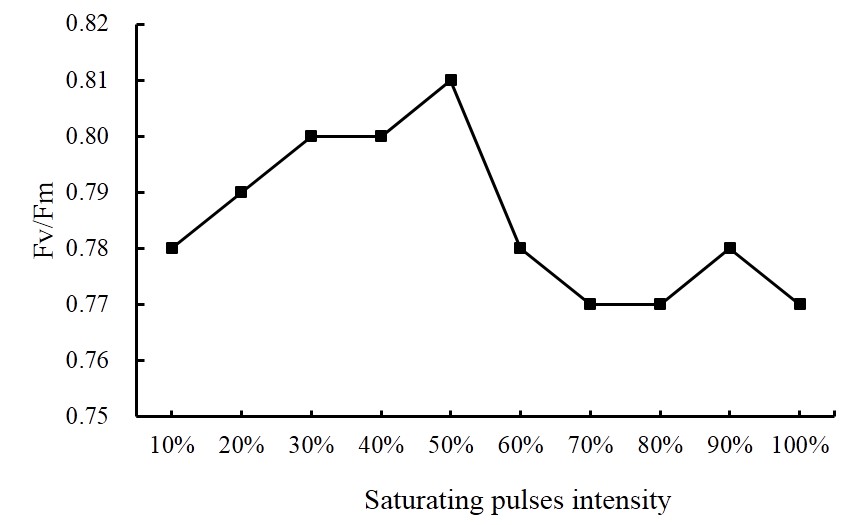
**

Figure S4. Maximum PSII quantum yield (*Fv/Fm*) measured at different intensities of the saturating pulses in the range of 10%-100%.
